# Validation of an e-health readiness assessment framework for developing countries

**DOI:** 10.1186/s12913-020-05448-3

**Published:** 2020-06-23

**Authors:** Kabelo Leonard Mauco, Richard E. Scott, Maurice Mars

**Affiliations:** 1 Department of TeleHealth, University of KwaZulu-Natal, Durban, South Africa; 2 Botho University, P O BOX 501564, Gaborone, Botswana; 3 NT Consulting - Global e-Health Inc, Calgary, Alberta Canada; 4 Department of Community Health Sciences, University of Calgary, Calgary, Alberta Canada; 5 Flinders University, Adelaide, South Australia Australia

**Keywords:** E-Health, E-health readiness assessment, Frameworks, Models, Validation, Botswana, Developing countries

## Abstract

**Background:**

Studies document e-health as having potential to improve quality of healthcare services, resulting in both developed and developing countries demonstrating continued interest in e-health uptake and use. e-Health implementations are not always successful as high failure rates have been reported in both developed and developing countries. These failures are often a result of lack of e-health readiness. e-Health readiness has been defined as the preparedness of healthcare institutions or communities for the anticipated change brought by programs related to information and communication technologies. As such it is critical to conduct an e-health readiness assessment prior to implementation of e-health innovations so as to reduce chances of project failure. Noting the absence of an adequate e-health readiness assessment framework (eHRAF) suitable for use in developing countries, the authors conceptualised, designed, and created a developing country specific eHRAF to aid in e-health policy planning. The aim of this study was to validate the developed eHRAF and to determine if it required further refinement before empirical testing.

**Methods:**

Published options for a framework validation process were adopted, and fifteen globally located e-health experts engaged. Botswana experts were engaged using saturation sampling, while international experts were purposively selected. Responses were collated in an Excel spreadsheet, and NVivo 11 software used to aid thematic analysis of the open ended questions.

**Results:**

Analysis of responses showed overall support for the content and format of the proposed eHRAF. Equivocal responses to some open ended questions were recorded, most of which suggested modifications to terms within the framework. One expert from the developed world had alternate views.

**Conclusions:**

The proposed eHRAF provides guidance for e-health policy development and planning by identifying, in an evidence based manner, the major areas to be considered when preparing for an e-health readiness assessment in the context of developing countries.

## Background

e-Health involves a broad group of activities that use electronic means to deliver health related information, resources and services [1]. The World Health Organization (WHO) has therefore defined e-health as the use of information and communication technologies (ICT) for health [2]. e-Health has also been referred to as forms of prevention and education, diagnostics, therapy and care delivered through digital technology independently of time and place [3]. As such e-health has the potential to improve quality of healthcare services [4], resulting in at least 125 developed and developing countries demonstrating continued interest in e-health uptake and use [5, 6].

Motivation to embrace e-health in developed versus developing countries will differ based on the needs and circumstances at play in a particular country classification. Adoption priorities for e-health in developing countries are likely to be influenced by significant health workforce shortages [7], shortage of resources (e.g. infrastructure, medical, and financial resources) as well as struggles with both communicable and non-communicable disease [8]. In developed countries e-health focuses on applying ICT to improve the efficiency and effectiveness of healthcare delivery, while in emerging and developing countries focus is directed towards providing access to basic healthcare for people [9].

Even with all its promises, e-health implementations are not always successful and implementation challenges have been documented. Historically failure rates of up to 70% have been reported [10] and many e-health interventions have been reported to fail during clinical implementation [11], to sputter along [12], to be plagued by expensive problems [13], or to be regarded as spectacular [14] or even dangerous failures [15]. Particular challenges associated with e-health implementation in developing countries include; lack of skilled stakeholders, inadequate infrastructures, lack of acceptance, limited resources, inadequate information communication as well as inadequate process guidance [16].

Case studies from developed countries are instructive, with many reasons identified as contributors to failure of an e-health project implementation [14, 17]. The UK’s National Programme for IT (NPfIT) was reported to lack both adequate end user engagement and trust, as well as lack of a phased change management approach. In addition it suffered from enforced top down decisions, and its profound scale was underestimated [14]. In Germany, a failed e-prescription implementation had many reported failings: too technical and design focused, insufficient in focus on processes and organisational change, lacking in an overall and integrated architecture at the outset (which was not developed over time), and including no appropriate time frames for development projects. Assessments during the course of the interventions were not taken into account and governance structures were unclear and changed over time. Furthermore, the opposition of the national medical assembly was not taken seriously (the project required additional time commitments from physicians who would not profit from the service) and should not have been implemented. In addition, any benefit from nearly simultaneous starts of field test projects was unclear, there was inflexibility regarding the re-prioritisation of tasks, and insufficient time to deeply test the technology in the laboratory. Although a cost-benefit analysis was conducted, it was performed too late and not published, and showed a negative cost-benefit for five and ten year projections [17].

These examples emphasise that failure of an e-health implementation may have nothing to do with the e-health technology employed, but rather may be due to a lack of preparedness of the setting (healthcare institutions or communities) in which the e-health innovation is to be implemented. Reasons for lack of such preparedness in both developed and developing countries may generally be similar but may also differ based on unique circumstances present in any given country. The term e-health readiness has been used to describe the preparedness of healthcare institutions or communities for the anticipated change brought by programs related to ICT [18]. As such it is critical to conduct an e-health readiness assessment prior to implementation of e-health innovations so as to reduce the chances of project failure. An e-health readiness assessment represents a significant step to analysing the existing setting and providing appropriate approaches to successful e-health transformation [19].

**Fig. 1:**
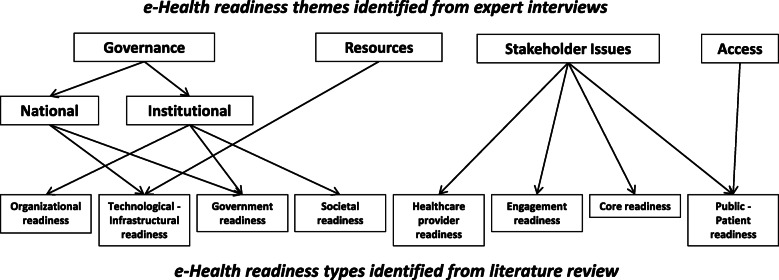
Mapping of e-health readiness themes identified by experts to e-health readiness types from literature [21]

**Fig. 2:**
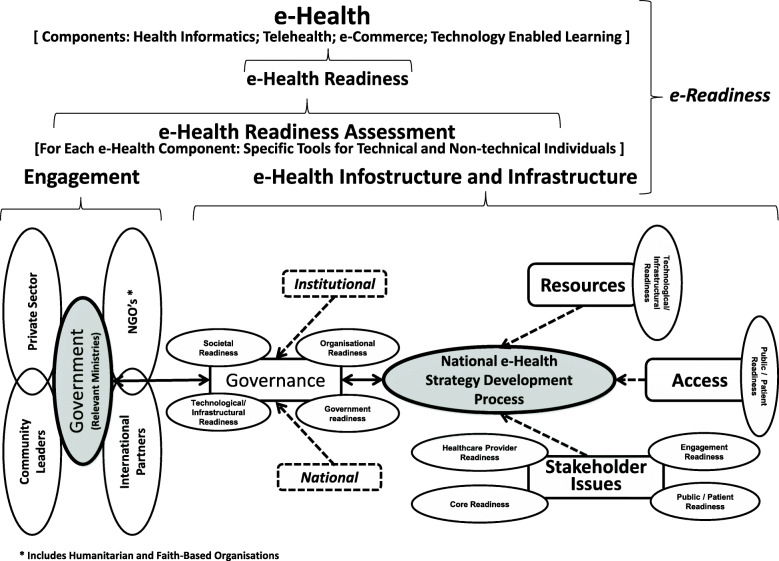
e-Health readiness assessment framework for developing countries (Mauco KL, Scott RE, Mars M: Development of a conceptual framework for e-health readiness assessment in the context of developing countries, submitted)

A previous study by the authors documented the existence of various e-health readiness assessment tools and frameworks in the literature, of which none were found entirely suitable nor adequate on their own to assess e-health readiness in the context of developing countries [20]. Based on this work, a subsequent study developed consistency in defining e-health readiness assessment themes and e-health readiness types based upon e-health expert’s opinion and the literature. This is illustrated in Fig. 1 [21]. This then led to conceptualisation and development of an e-health readiness assessment framework (eHRAF) suitable for use in developing countries (Fig. 2).

The definitions of each e-health readiness type are as follows [21];

Organisational Readiness: The extent to which the institutional setting and culture supports and promotes awareness, implementation, and use of e-health innovations (e.g., presence of relevant policies; senior management support).

Technological / Infrastructural Readiness: The availability and affordability of ICT resources necessary to implement a proposed e-health innovation (e.g., skilled human resources, ICT support, quality ICT infrastructure, and power supply).

Government Readiness: The extent to which a country’s Government and politicians support and promote awareness, implementation, and use of e-health innovations (e.g., presence of relevant policies, and funding.

Societal Readiness: The degree of ‘interaction’ associated with a healthcare institution. Interaction is described by three parameters; interaction among members of a healthcare institution, interaction of a healthcare institution with other healthcare institutions, and interaction of a healthcare institution with its local communities.

Healthcare Provider Readiness: The influence of a healthcare provider’s personal experience; primarily their perception and receptiveness towards the use of e-health technology.

Engagement Readiness: The extent to which members of a community are exposed to the concept of e-health and are actively debating its perceived benefits as well as negative impacts. It also involves gauging the willingness of members of a community to accept training on e-health.

Core Readiness: The extent to which members of a community are dissatisfied with the current status of their healthcare service provision, see e-health as a solution, and express their need and preparedness for e-health services.

Public / Patient Readiness: The extent to which members of the public and patients are aware of, and can afford and access, e-health services. It also involves gauging the influence of their personal experiences on their perception and receptiveness towards the use of e-health technology.

The proposed e-health readiness framework for developing countries first illustrates the overarching role of ‘e-readiness’ of a setting which will inevitably impact ‘e-health readiness’ (Fig. 2). The framework then prompts assessment of e-health readiness to be conducted separately for each component of e-health (health informatics, telehealth, e-commerce, and technology enabled and enhanced teaching). In terms of actual assessment, the need for separate e-health readiness assessment tools for technical and non-technical individuals (e.g., ICT staff versus clinicians and managers) is illustrated [20, 21]. Two aspects are then identified as essential factors in determining an e-health ready setting, both of which require specific assessment; first, stakeholder engagement, and second, the presence of relevant e-health infostructure and infrastructure. Within the framework, government is at the core of stakeholder engagement.

Relevant and essential stakeholders to be engaged are; the private sector, community leaders, international partners, as well as non-governmental organisations (NGOs), humanitarian organisations, and faith based groups (Fig. 2). The need for development of a comprehensive and informed national e-health strategy as a prerequisite to e-health readiness is shown. Also highlighted are the four themes of governance (institutional and national), resources, stakeholder issues, and access. These were identified by experts in Botswana as important considerations during the development of the national e-health strategy and achieving e-health readiness. e-Health readiness assessment types (defined above) were extracted from the literature [20]. They are included in the framework and aligned to related themes from the expert interviews (Fig. 1), and are also considered to be important during development of a national e-health strategy and towards achieving e-health readiness.

It was necessary to validate the proposed framework. Whilst the literature shows validation of various e-health readiness ‘tools’ [22, 23] there is no specific guidance for validation of e-health readiness ‘frameworks’. ‘Validation’ can be undertaken in several ways. Some approaches have involved psychometric assessment [24] or face and content validation [25]. Others have used broad opinion gathered through social media (Facebook and LinkedIn) and either self-administration of a survey or interviews with a sample of users [26]. In examining how to validate quality frameworks in e-learning, six approaches to validation were identified, including review of appropriate research literature, undertaking survey research, and using the knowledge of experts in the field [27]. Overall, no specific and accepted approach to validation of e-health related frameworks has been explicitly described. Here, validation is considered the process of establishing evidence that confirms the eHRAF will be capable of consistently guiding the process it is supposed to, and that it meets the operational needs of the planned users. Considering the work of Inglis (2008) and because the literature had already been used in the design process, a survey approach to seek the opinion of international experts as well as planned users within Botswana was considered the appropriate route to validate the eHRAF. The aim of this study was, therefore, to validate the aforementioned e-health readiness assessment framework through expert survey and to determine if the framework required further refinement before empirical testing.

## Methods

Validation involved sending a questionnaire to 15 e-health experts. The experts comprised six of the 18 Botswana e-health experts whose input had contributed to the development of the eHRAF, three e-health experts from low and middle income countries within sub-Saharan Africa (LMIC within SSA), three e-health experts from low and middle income countries outside sub-Saharan Africa (LMIC outside SSA), and three e-health experts from the developed world. Selecting six out the previously engaged 18 Botswana e-health experts was based on the principle that, in qualitative research, more data does not necessarily lead to more information [28] and that data collection and analysis should continue only until no new concepts emerge [29]. Hence the number of Botswana e-health experts included in this study was based on ‘data saturation’, involving iterative analysis of responses from sets of consecutively sampled groups of experts within Botswana (two per group) until no new concepts or patterns were generated. An ‘initial sample size’ of four was adopted, with the ‘stopping criterion’ being a progressive sampling of two more individuals with re-analysis until data saturation was reached [30]. International experts were purposively selected based on their e-health experience and expertise.

Potential participants were initially invited by email, and after indicating their interest in participating, they were sent a formal letter of invitation, a consent form, the developed eHRAF (Fig. 2), explanatory notes, and the self-administered questionnaire. One of the selected e-health experts from the developed world who had originally agreed to participate failed to respond to the questionnaire and was replaced with another from the developed world.

Explanatory notes detailed the purpose of the framework and also included the definitions of e-health readiness assessment themes and e-health readiness types as applied to the framework (Fig. 1). This was done to engender consistency and guide the experts through the framework validation process. The questionnaire contained 11 statements using a six point Likert scale, and 14 open-ended questions. The Likert scale statements sought opinion on the fundamental aspects that guided the framework design, and did not have the option for free text responses. The open-ended questions sought opinion on the framework layout and whether the framework met its intended purpose.

Preliminary pilot testing of the questionnaire was undertaken with 10 people prior to distribution to determine if there was confusion or ambiguity with any statement or questions. The survey statements and questions were refined where necessary. Responses from all the experts were collated in an Excel spreadsheet and NVivo 11 software was used to aid thematic analysis of responses to the open-ended questions.

## Results

Analysis of responses to closed-ended statements (Table 1) and to open-ended questions (Table 2) showed overall support for the content and format of the proposed eHRAF. The median score for all statements (Table 1) was 5 or 6, where 6 represented ‘strongly agree’. Individual responses were positive, with only two respondents having a median score of less than 5, both experts were from the developed world. Of the 165 responses to the close-ended statements, disagreement with a statement was noted in only 12 responses (7.3%). Two respondents, both from the developed world, accounted for 9 of the 12 differences in opinion. The maximum disagreement with any statement was two respondents for three statements. These statements related to the need for separate assessment tools and the stakeholder roles of the private sector and other organisations (e.g., NGOs).

**Table 1:** Validation experts’ opinion on the fundamental concepts that guided the framework design

Statements	Likert scale responses^a^ from globally located experts															Median score
	Botswana						LMIC within SSA			LMIC outside SSA			Developed world			
1. Overall ‘eReadiness’ of a setting has an impact on its ‘ehealth readiness’.	4	6	5	6	5	6	5	5	6	6	5	6	5	6	6	6
2. Assessment of ehealth readiness needs to be conducted separately for each component of ehealth (health informatics, telehealth, ecommerce, and technology enabled learning).	4	6	5	5	4	5	4	6	**2**	5	5	6	5	6	5	5
3. Use of separate ehealth readiness assessment tools is necessary for technical and nontechnical individuals (e.g., ICT staff versus clinicians or managers).	5	6	5	6	5	5	5	6	**2**	5	4	6	5	**2**	4	5
4. Stakeholder engagement is a prerequisite for ehealth readiness.	5	6	6	6	5	6	6	6	6	6	6	6	6	6	6	6
5. Government needs to be at the core of the stakeholder engagement process.	5	5	6	6	4	4	6	6	6	5	6	6	4	**1**	4	5
6. The private sector is an essential stakeholder to be engaged.	5	5	6	6	5	6	6	6	5	6	6	5	5	**3**	**3**	6
7. Community leaders are an essential stakeholder to be engaged.	5	5	6	6	5	5	6	6	5	6	6	5	5	**3**	5	5
8. International partners are an essential stakeholder to be engaged.	4	5	5	6	5	5	6	6	5	6	5	**3**	5	5	**3**	5
9. NGOs, humanitarian organisations, and faith based groups are an essential stakeholder to be engaged.	5	5	5	6	5	5	5	6	5	5	5	5	5	4	**2**	5
10. Presence of relevant ehealth infostructure and infrastructure are a prerequisite for ehealth readiness.	6	6	5	4	6	6	4	6	6	6	6	6	5	**1**	4	6
11. Presence of a national ehealth strategy is a prerequisite for ehealth readiness.	6	5	5	4	5	6	4	6	6	5	6	6	4	**1**	4	6
**Median overall score response from each expert**	5	5	5	6	5	5	5	6	5	6	6	6	5	**3**	4	

^a^Six point Likert scale; From 1 = strongly disagree to 6 = strongly agree

**Table 2:** A summary of validation experts’ responses to suggested modifications to the framework; open-ended responses

Questions	Responses from globally located experts														
	Botswana						LMIC within SSA			LMIC outside SSA			Developed world		
1. Considering the narration given with regards to governance, is this theme rightfully placed in the framework?	+	+	+	+	+	+	+	+	e	+	+	+	+	+	+
2. Considering the narration given with regards to stakeholder issues, is this theme rightfully placed in the framework?	+	+	+	+	+	+	+	+	e	+	+	+	+	e	e
3. Considering the narration given with regards to resources, is this theme rightfully placed in the framework?	+	+	+	+	+	+	+	+	+	+	+	+	+	+	+
4. Considering the narration given with regards to access, is this theme rightfully placed in the framework?	+	+	+	+	e	+	+	+	e	+	+	+	+	e	+
5. Considering the narration given with regards to organisational readiness, is this e-health readiness type rightfully placed in the framework?	+	+	+	+	+	+	+	+	e	+	+	+	+	e	+
6. Considering the narration given with regards to technological/infrastructural readiness, is this e-health readiness type rightfully placed in the framework?	+	+	+	+	+	+	+	+	e	+	+	+	+	e	+
7. Considering the narration given with regards to healthcare provider readiness, is this e-health readiness type rightfully placed in the framework?	+	+	+	+	e	+	+	+	e	+	+	+	+	e	+
8. Considering the narration given with regards to engagement readiness, is this e-health readiness type rightfully placed in the framework?	+	+	+	+	e	+	+	+	+	+	+	+	+	e	e
9. Considering the narration given with regards to societal readiness, is this e-health readiness type rightfully placed in the framework?	+	+	+	+	e	+	+	+	+	+	+	+	+	e	+
10. Considering the narration given with regards to core readiness, is this e-health readiness type rightfully placed in the framework?	+	+	+	+	e	+	+	+	+	+	+	+	+	e	e
11. Considering the narration given with regards to government readiness, is this e-health readiness type rightfully placed in the framework?	+	+	+	+	e	+	+	+	e	+	+	+	+	+	+
12. Considering the narration given with regards to public/patient readiness, is this e-health readiness type rightfully placed in the framework?	+	+	+	+	e	+	+	+	+	+	+	+	e	e	e
13. Considering the intent of the framework proposed, which is to guide e-health readiness assessment in the context of developing countries (using Botswana as the exemplar), and considering the proviso that specifics of the content must be modified to be context (country) specific: Is the framework in its current form suitable to achieve this?	+	+	+	+	+	+	+	+	e	+	e	+	e	–	e
14. Do you believe the eHRAF (as modified based upon expert validation by you and others) will be a useful tool for developing countries?	+	+	+	+	+	+	+	+	+	+	+	+	+	–	+

+ = Agreement; − = Disagreement; e = Equivocal

Of the 210 open-ended responses from validation of the framework’s constructs and layout, 176 (84%) responses were positive, only two (1%) of responses showed differences of opinion (both were from one expert from the developed world), and 32 (15%) of responses were considered equivocal (Table 2). The negative responses were for questions 13 and 14. Question 13 posited “Considering the intent of the framework proposed, which is to guide e-health readiness assessment in the context of developing countries (using Botswana as the exemplar), and considering the proviso that specifics of the content must be modified to be context (country) specific: Is the framework in its current form suitable to achieve this?” and the response was “I guess all my comments illustrate what is needed in my opinion”. In turn, question 14 posited “Do you believe the eHRAF (as modified based upon expert validation by you and others) will be a useful tool for developing countries?” and the response was “Not as is - it would be another generic, unspecified tool without empirical testing and proof of its validity”.

‘Equivocal responses’ were those that had nothing to do with an expert rejecting the framework, but more to do with suggesting some modifications to the framework. Suggested modifications included; renaming some of the e-health readiness assessment types and themes (two experts from the developed world and one from Botswana), a need to make the framework sector specific (two from the developed world), removal of ecommerce from the framework (one from LMIC within SSA), addition of some constructs to the framework (one expert each from; LMIC within SSA, the developed world, Botswana, and LMIC outside SSA), and strengthening the navigation utility of the framework (one from LMIC within SSA). Although respondents agreed with the content the remaining 21 equivocal responses were often related to positioning of an accepted element within the framework (although they agreed with its inclusion), were generic statements (e.g., ‘It’s a title not a theme’, ‘Access must be linked with usage’), or were comments whose intent was felt to be already reflected within the framework (e.g., ‘Readiness differs, depending on setting (country, local level etc.)’, ‘Certainly you need to define the ehealth service to which this applies, as the readiness will vary’).

Some experts provided alternate suggestions contrary to the stated purpose of the proposed framework. For example; “readiness of a country, a region, a local situation, a single hospital, a single GP office or community centre needs different tools for assessment”, “depending on the institutional level (national versus GP office) readiness concerned totally different elements”, “[it is] unclear as to whom healthcare provider specifically refers to”, “do not see an obvious way that the framework can be used, perhaps a description of that would help me evaluate it better.” This is despite being provided with Fig. 1 as well as statements explaining the purpose of the proposed eHRAF in the explanatory notes sent to the experts.

A few comments also suggest that one expert only envisioned a one to one relationship between certain framework constructs/concepts, disregarding their overall role in the framework. Some of these comments included; “I agree with the comments and items listed for stakeholders. In the Figure I am not sure that the bubbles on the left (government and the other large stakeholder groups) should be separated from the Stakeholder Issues box. I wonder whether the engagement process on the left will be the process through which the issues on the right are brought into the strategy process. If that is the case, then the Figure may need some reorientation to show this”, “I agree with the broad perspective you have taken on Access. I would like to see a closer relationship between Access and connectivity, which I presume sits within Resources, since without connectivity being addressed, there is no real access at all.”, “I see organizational readiness as having elements of all four themes above, and find that where it is currently placed in the Figure makes it too tightly coupled to Governance alone.” Appendix 1 shows this in more detail (see Additional file 1).

Selection of Botswana experts was based on application of the data saturation method, as previously explained. When comparing analysis of the first four and then six experts, the iterative, sequential analysis showed no marked variation between open-ended responses; all the Botswana experts’ responses were favourable, with only one suggesting an alternate title for a ‘theme’. Furthermore their Likert scale responses did not differ (all were positive, ranging from ‘4’ to ‘6’ with each posited statement) (Table 1).

## Discussion

In the absence of comparative literature regarding validation of e-health readiness assessment frameworks, the discussion focusses on a review and critique of the findings of the validation study. Overall there was significant support for the framework, with only one expert from the developed world showing differences of opinion (open-ended questions) or scoring (closed-ended questions). There were only four experts out of the fifteen who disagreed with some fundamental aspects that guided the framework design; i.e., where the experts’ responses were 3 or less (Table 1). There were only three closed-ended responses (questions 3, 6, and 8) where more than one expert disagreed with the statement (two experts from the developed world, one expert from LMIC within SSA and one expert from LMIC outside SSA) (Table 1). No more than two respondents disagreed with any statement. As such, there was no framework construct with which the experts entirely disagreed, as also illustrated by the experts’ responses to the open-ended questions (Table 2). Some experts seem to have overlooked the explanatory notes that accompanied the survey tool and this may be the reason for some of the alternate suggestions.

Several comments from some experts related to re-wording of themes or readiness types. As an example, Question 8 in the open-ended section of the questionnaire posited “Considering the narration given with regards to engagement readiness, is this e-health readiness type rightfully placed in the framework?” and a response was “engagement readiness sound a bit too difficult for anybody to process, I will suggest you call it Public readiness.” However, a framework has to be evidence based. The proposed eHRAF deliberately attempted to unify and provide consistency in use and definitions of e-health readiness themes and types. As a consequence the authors refrained from altering the e-health readiness assessment themes and types used in the eHRAF.

One expert suggested that e-commerce be removed from the framework. e-Commerce refers to the use of ICTs to conduct business transactions among buyers, sellers, and other trading partners, as well as mechanisms for reimbursement of healthcare providers for services provided. No matter the country, healthcare incurs ‘costs’ typically paid for through one or more of government, insurers, NGOs, or consumers. To patients or consumers, the costs are usually what they pay out of pocket for healthcare services or insurance premiums. To healthcare providers (healthcare organisations or clinicians) the costs relate to expenses they incur in delivering services (and can include items such as equipment, depreciation, personnel, and overhead) [31]. Literature has documented e-commerce as a critical and integral component of e-health [32–34] and should be a component of eHRAF.

One expert expressed the view that the concept of culture had to be represented in the framework. Culture can be loosely defined as a way of life of a group of people (including both communities and organisations). The following eHRAF constructs, as well as their definitions, illustrate that aspects of culture are considered within the framework; societal readiness, organisational readiness, government readiness, public\patient readiness, engagement readiness, and core readiness. As a consequence whilst not overtly identified, culture is represented within the proposed framework. Future iterations will clarify the above to ensure culture is more clearly embedded.

In response to closed-ended questions one expert from the developed world, disagreed with the purpose and context of the framework (Table 1). Greatest disagreement was for questions 5, 10, and 11, where this was the only dissenting opinion. Question 5 posited that government needs to be at the core of the stakeholder engagement process, question 10 posited that relevant e-health infostructure and infrastructure are pre-requisites for e-health readiness, and question 11 posited that a national e-health strategy is a pre-requisite for e-health readiness. Given that governments are the entities with sovereign responsibility to deliver healthcare, it would make sense that they should be central to engaging those who will use the e-health services they endorse or provide. Furthermore, it is difficult to conceive of a setting to be ready to deliver e-health services without relevant e-health infostructure and infrastructure. Finally, in regard to e-health strategy the World Health Organization and other authors have emphasised the critical role of an e-health strategy in e-health implementation [35–37]. The framework presented recognises e-health strategy as a core component to ensure readiness. Ironically, many of the issues raised by this and other experts regarding the framework could be resolved through preparation of a sound e-health strategy that considers and rationalises which e-health solutions are practical and appropriate for a country. Such an e-health strategy should be founded upon an evidence based situational assessment, and recognise the evidence based health and healthcare needs for the setting.

### eHRAF application

Introducing and using a new framework may be perceived to add layers of complexity to a task that, in most developing countries, is poorly performed or not undertaken at all. The intricacy and opportunity costs of e-health implementations, and their ongoing maintenance and sustainability costs, cannot be disregarded. Ensuring e-health readiness prior to a healthcare facility or country committing to e-health implementations is critical and requires real-life application of the eHRAF. As a conceptual framework the eHRAF identifies and describes the integral role of, and inter-relationships between, the “main elements”, arranging them in a logical structure to provide a picture or visual display of how ideas relate to one another [38, 39]. The elements must be interpreted both in the totality of the framework and how they interact within the framework, and not in isolation; changes in one element will have ripple effects elsewhere. Considered in this fashion, the eHRAF guides by noting and relating the main aspects of e-health readiness to be considered for a thorough e-health readiness assessment within a developing country. These main elements will be familiar, being drawn from the existing e-health readiness literature, thereby reducing complexity and easing practical application of the framework. However, the framework is not proscriptive in its application in the sense of proceeding from left to right or from top to bottom. The eHRAF is also not detailed, since the specific circumstances at play in any particular setting will dictate where focussed effort is required. Examining Fig. 2 and reflecting on the reality of a setting in regard to the maturity of each of the main elements will reveal areas of strength, weakness or absence of consideration. These can then be tackled accordingly (maintenance, strengthening, or development; respectively), whilst ensuring concomitant response to all other elements remains aligned in their progression and completion.

### Limitations

One goal of the validation process was to determine if the eHRAF met the operational needs of the planned users, requiring validation by Botswana experts. However, this represents a potential limitation of the study, since the Botswana respondents had been a part of the design process and may have biased opinion. In addition the validation process does not provide insight into utility in practice, which will require further empirical testing.

## Conclusions

A national eHRAF provides an opportunity for a country to assess its readiness to adopt e-health; an essential component in the policy development and planning process for e-health. An eHRAF would thus inform the e-health implementation process, hence reducing implementation failure rates. Realising the absence of an eHRAF in the context of developing countries, the authors have filled this gap. The eHRAF presented is intentionally generic in its design. It provides guidance by identifying, in an evidence based manner, the major areas to be considered when preparing for an e-health readiness assessment in the context of developing countries. The framework will also guide development of the relevant assessment tools to be used during the e-health readiness assessment process. As previously noted, particular circumstances at play in any specific developing country may require adaptation of the framework to suit the specific setting and process. For example, future empirical research needs to confirm whether the relevant owner of a national eHRAF is the government, as well as confirming the efficacy of the eHRAF in ensuring successful implementation of e-health.

## Supplementary information

**Additional file 1:** Appendix 1: Validation experts’ suggested modifications to the framework; open ended responses. Open ended responses to a validation questionnaire by e-health experts.

## Data Availability

The data collected and analysed during the current study is available from the corresponding author on reasonable request.

## Abbreviations

eHRAF: e-Health readiness assessment framework(s)
ICT: Information and communications technologies
LMIC: Low and middle income countries
SSA: Sub-Saharan Africa
NGOs: Non-governmental organisation(s)
