# Supplementary material for: Validation of an e-health readiness assessment framework for developing countries
Source: BMC Health Serv Res. 2020 Jun 23;20:575. doi: 10.1186/s12913-020-05448-3 (PMC7313186; doi:10.1186/s12913-020-05448-3)
Supplement: Supplementary file 1 — Additional file 1: Appendix 1: Validation experts’ suggested modifications to the framework; open ended responses. Open ended responses to a validation questionnaire by e-health experts. [file 12913_2020_5448_MOESM1_ESM.docx]

| Question | Suggested modification to the framework | Expert |
| --- | --- | --- |
| 1. Considering the narration given with regards to governance, is this theme rightfully placed in the framework? | 1. I agree with the importance of a governance framework. What I don’t agree with is the positioning of governance in your figure, where it sits between Government (and other stakeholders) and the eHealth Strategy. Rather, I see it as a core component of all aspects of the readiness framework, both as an input that guides all interactions (by setting the “rules of the game” at the outset) and also as an outcome of the strategy, since all component of the strategy will require governance development, whether it be how stakeholders interact, oversight over how resources are deployed and access is managed, or more specialised governance aspects such as data governance. I wonder whether the bi-directional arrow is meant to represent that governance shapes the strategy as well as is an output of the strategy, though it is not clear to me. | LMIC within SSA |
| 2. Considering the narration given with regards to stakeholder issues, is this theme rightfully placed in the framework? | 1. Stakeholder issues is a title and not a theme. You would identify specific issues as the themes for inclusion in the eHRAF and not a box labelled "issues", as they may be several separate issues | Developed world |
|  | 2. I agree with the comments and items listed for stakeholders. In the figure I am not sure that the bubbles on the left (government and the other large stakeholder groups) should be separated from the Stakeholder Issues box. I wonder whether the engagement process on the left will be the process through which the issues on the right are brought into the strategy process. If that is the case, then the figure may need some re-orientation to show this. A related question I have is around how health strengthening/transformation is reflected in your figure. This is often the key output that stakeholders require (i.e. some fundamental change in how the health system works) | LMIC within SSA |
|  | 3. This is too simplistic an approach: there are very different types of involvement(from just informing after the fact to a direct say in planning and policy development decisions; and different stakeholders differ in relevance-e.g MDs are often "veto players" | Developed world |
| 3. Considering the narration given with regards to resources, is this theme rightfully placed in the framework? | 1. I am comfortable with where you place resources. I wonder about whether. it is necessary to reflect the relationship between inputs and outputs (a broad way of looking at the total societal costs and benefits) to ensure that the particular country or system has an understanding of the importance of assessing cost-benefit and ensuring that it generates a net benefit before investing public funds in an eHealth initiative | LMIC within SSA |
|  | 2. Differentiate between resources available, resources needed but not available, however which may be created in a short while; resources needed but not available within a given time horizon, resources very useful but not essential etc | Developed world |
| 4. Considering the narration given with regards to access, is this theme rightfully placed in the framework? | 1. I believe the access has to always go with usage, access will always make the service available, but it is important to consider issue of usage and how in the framework people will be motivated or incentivized to use the service | Botswana |
|  | 2. I agree with the broad perspective you have taken on Access. I would like to see a closer relationship between Access and connectivity, which I presume sits within Resources, since without connectivity being addressed, there is no real access at all. | LMIC within SSA |
|  | 3. Access to patient and other medical, administrative, resources and health systems (public health) data is a highly complex issue which need not be considered for readiness assessment, BUT is the essence of all of this (The policy goal as the base for improving healthcare etc) i.e outside the framework. You mix up levels in the theoretical constructs here. You want to assess readiness for achieving a given level and domain of access, but this cannot be part of readiness | Developed world |
| 5. Considering the narration given with regards to organisational readiness, is this e-health readiness type rightfully placed in the framework? | 1. But can suggest it gets called Institutional governance, so that it is cross cutting including government readiness. | Botswana |
|  | 2. I see organizational readiness as having elements of all four themes above, and find that where it is currently placed in the figure makes it too tightly coupled to Governance alone. | LMIC within SSA |
|  | 3. Readiness of a country, a region, a local situation (e.g in a metropolitan area like Sao Paulo), a single hospital, a single GP office or community centre needs different tools for assessment, because the goal of the information needed for relevant decision taking differ, and the type of information/data needed differ. | Developed world |
| 6. Considering the narration given with regards to technological/infrastructural readiness, is this e-health readiness type rightfully placed in the framework? | 1. I see technological/infrastructure readiness as having elements of other themes (particularly Resources), and find that where it is currently placed in the figure makes it too tightly coupled to Governance alone. | LMIC within SSA |
|  | 2. Depending on the institutional level (national versus GP office) readiness concerns totally different element. | Developed world |
| 7. Considering the narration given with regards to healthcare provider readiness, is this e-health readiness type rightfully placed in the framework? | 1. This should be captured under organizational readiness, no healthcare provider will be operating in isolation. Reducing the number of assessment themes helps to enable participation | Botswana |
|  | 2. While I am happy with the overall placement of the provider for the specific purpose described above, I find that I would like to see a person (patient and health worker) more central in the figure. | LMIC within SSA |
|  | 3.unclear, whom you have in mind: healthcare provider’s personal experience- clearly differentiate organizational readiness (healthcare provider as organization like a hospital) from personal views and knowledge of individual healthcare professionals, nurses, health workers, patients, careers- and also IT staff. | Developed world |
| 8. Considering the narration given with regards to engagement readiness, is this e-health readiness type rightfully placed in the framework? | 1. Engagement readiness sound a bit too difficult for anybody to process, I will suggest you call it Public readiness | Botswana |
|  | 2. This needs clarification of "the community”. When applied to the public then there is little need for training and therefore acceptance. | Developed world |
|  | 3. I have difficulty with Engagement Readiness not being aligned with the Engagement part of the Figure. I can see partly why there is an engagement focus specific to the Strategy process, though I would prefer them to be aligned for the figure to make sense for me. | LMIC within SSA |
|  | 4. Concrete evidence indicates that you "only" need to engage the leaders (of the GP association. Leading doctors in a hospital, head nurses) sometimes if their peers trust them, then they will follow what their leaders decided for them- and successfully. | Developed world. |
| 9. Considering the narration given with regards to societal readiness, is this e-health readiness type rightfully placed in the framework? | 1. For the same argument raised earlier, this could just be under what I have proposed to be Public readiness | Botswana |
|  | 2. (Participant did not provide a YES or NO answer)-where are we here- at the national policy/strategy level, or the local level or? This again illustrates the inconsistency in the framework. | Developed world |
| 10. Considering the narration given with regards to core readiness, is this e-health readiness type rightfully placed in the framework? | 1. For the same argument raised earlier, this could just be under what I have proposed to be Public readiness. | Botswana |
|  | 2. This is very subjective, which needs consideration, as it will depend on their perception of the benefits of the e-health service. | Developed world |
|  | 3. This is a very negative view-most doctors always want to improve and are not necessarily dissatisfied with what they do and achieve. | Developed world |
| 11. Considering the narration given with regards to government readiness, is this e-health readiness type rightfully placed in the framework? | 1. Can I suggest it gets called Institutional governance, so that its cross cutting including government readiness | Botswana |
|  | 2. The relevance will depend on the scale of operation, e.g. is it to be national policy, or operated at local level | Developed world |
|  | 3. I can’t see why Government Readiness should not be linked to the Government bubble, rather than the Governance bubble, which seems too narrow a perspective to cover Government readiness the way it is defined above. | LMIC within SSA |
|  | 4. The role of government and its impact depends on the type of healthcare systems, and at the political level it may be even more important for parliament to lead and tell the government what to do. | Developed world |
| 12. Considering the narration given with regards to public/patient readiness, is this e-health readiness type rightfully placed in the framework? | 1. This should be components under the larger Public readiness proposed above | Botswana |
|  | 2. Some overlap between public/patient, core readiness and engagement readiness | Developed world |
|  | 3. The relevance will depend entirely on the e-health service under consideration. Generally the public/patient accept what is given by the clinician if they are convinced it is beneficial | Developed world |
|  | 4. I am very happy to see the patient explicitly mentioned, though I would prefer to see a relationship between this and other parts of the Figure, particularly Access. | LMIC within SSA |
|  | 5. Too simple: you need a certain level of IT savvy of patients, and of course electricity and telecommunication access, but you also need doctors and nurses whom they trust and who explain what digital health can do-if they trust them, they will follow them, or not. But there are many, probably more important initial applications where patients are not at all involved as users. Again, it depends on health policy priorities and goals. | Developed world |
| 13.Considering the intent of the framework proposed, which is to guide e-health readiness assessment in the context of developing countries (using Botswana as the exemplar), and considering the proviso that specifics of the content must be modified to be context (country) specific: Is the framework in its current form suitable to achieve this? | 1. The only potential improvement to consider would be refining the relationship between public patient readiness, core readiness and engagement readiness. | Developed world |
|  | 2. Some changes might be considered. Certainly you need to define the e-health service to which this applies, as the readiness will vary. E.g. teleclinics can be established in main centres but not to remote areas due to the difference in availability of telecommunication. i also believe that an analysis of this type also has to investigate the patient care pathway to determine whether the e-health service is appropriate for the circumstances and how it will integrate to existing healthcare services. e.g you can provide an e-health service to a remote clinic, but they may always refer to hospital and it may not be used. Infrequent use can prevent uptake. | Developed world |
|  | 3. But it's very important to move e-commerce from the framework. | LMIC within SSA |
|  | 4. I don’t see an obvious way that I would “use” the framework. Perhaps a description of that would help me evaluate it better. I would also like to see the relationships I have clarified above addressed in some way. | LMIC within SSA |
|  | 5. Culture needs to be included in the framework, perhaps as the 5th theme, as it plays a major role in both the readiness as well as implementation strategy. | LMIC outside SSA |
|  | 6. In the context of Botswana I would say yes, with modifications provided, the assesses are generally discouraged by large documents and it’s more prudent to streamline the assessment. | Botswana |
|  | 7. I guess all my comments illustrate what is needed in my opinion. | Developed world |
| 14. Do you believe the eHRAF (as modified based upon expert validation by you and others) will be a useful tool for developing countries? | 1. Readiness is an important issue and I believe developing countries will appreciate a tool that helps them to navigate the complex issues. This will require the eHRAF to be revised to strengthen its navigation utility. | LMIC within SSA |
|  | 2. Not as is-it would be another generic, unspecified tool without empirical testing and proof of its validity. | Developed world |
